# Assessing brain state and anesthesia level with two-photon calcium signals

**DOI:** 10.1038/s41598-023-30224-8

**Published:** 2023-02-23

**Authors:** Núria Tort-Colet, Francesco Resta, Elena Montagni, Francesco Pavone, Anna Letizia Allegra Mascaro, Alain Destexhe

**Affiliations:** 1 Paris-Saclay University, CNRS, Institut des Neurosciences (NeuroPSI), Saclay, France; 2 European Laboratory for Non-Linear Spectroscopy (LENS), Sesto Fiorentino, Italy; 3 Department of Physics and Astronomy, University of Florence, Sesto Fiorentino, Italy; 4 National Institute of Optics, National Research Council, Sesto Fiorentino, Italy; 5 Neuroscience Institute, National Research Council, Pisa, Italy

**Keywords:** Computational neuroscience, Neural circuits, Sensorimotor processing, Neuroscience

## Abstract

Brain states, such as wake, sleep, or different depths of anesthesia are usually assessed using electrophysiological techniques, such as the local field potential (LFP) or the electroencephalogram (EEG), which are ideal signals for detecting activity patterns such as asynchronous or oscillatory activities. However, it is technically challenging to have these types of measures during calcium imaging recordings such as two-photon or wide-field techniques. Here, using simultaneous two-photon and LFP measurements, we demonstrate that despite the slower dynamics of the calcium signal, there is a high correlation between the LFP and two-photon signals taken from the neuropil outside neuronal somata. Moreover, we find the calcium signal to be systematically delayed from the LFP signal, and we use a model to show that the delay between the two signals is due to the physical distance between the recording sites. These results suggest that calcium signals alone can be used to detect activity patterns such as slow oscillations and ultimately assess the brain state and level of anesthesia.

## Introduction

Anesthesia levels and brain states are classified according to the main features of global dynamics such as the frequency of the slow oscillations or the duration of the Up-state and Down-state^12,37^. Electrophysiological methods are commonly adopted to categorize brain states and to investigate the features of neural activation underlying a specific brain state over multiple scales. In particular, local field potential (LFP) is used to record both single unit and small population activity nearby the electrode, whereas electrocorticogram (ECoG) and electroencephalogram (EEG) are exploited to study neuronal activity dynamics at a larger scale. However, the electrophysiological recordings are poorly defined in terms of the type and spatial distribution of the cell originating the signal. To record the activity of up to hundreds of neurons simultaneously with cellular and subcellular resolution, Two-photon (2P) fluorescence microscopy is increasingly adopted in neuroscience laboratories^28^. This technique has been applied to the study of neuronal activity dynamics during sleep and anesthesia^7,40^. In particular, it has been demonstrated that the calcium activity from the neuropil of the cortical layer 2/3 is highly correlated with the electrical (ECoG) signal^19^. This pioneering study took advantage of 2P calcium imaging coupled with the calcium dye OGB-1-AM. Calcium dyes provide unspecific labeling of all types of neurons and astrocytes, thus limiting the possibility of identifying the cellular origin of the recorded optical signal.

To overcome this limitation, population-specific labeling of the neurons by genetically encoded calcium indicators (GECI) is increasingly adopted^18,30^. For instance, activity recordings of GECI via fiber photometry revealed a cortex-wide BOLD signal correlated with the local slow calcium wave signal^1,29^. In combination with GECIs, 2P fluorescence microscopy enables visualizing the activity of specific neuronal populations with subcellular resolution. Recent studies in rodents including^20,31^ in brain slices or^4,39^ *in vivo*, compared neuronal activity measured via electrophysiology recordings and 2P calcium imaging of small networks, to characterize the transform between neural activity and calcium-related fluorescence. This combination of techniques can provide valuable insight into how information on the brain state is represented and processed in specific neural circuits^15,24–27,33^. However, these studies rely on the electrical (electromyographical or EEG) signal as background information to define the brain state. It would be useful for the neuroscience and neuroimaging communities to use the 2P fluorescence signal to define the brain state and not rely on simultaneous electrical registrations, which are technically hard to arrange in most experimental setups. It is very important to combine different measurement methods to validate findings independently of the technique used. However, performing such comparisons is not a trivial task and, so far, only rarely addressed.

Because the neuropil volume is dominated by axons, dendrites, and synapses, and since the LFP is thought to be generated essentially from synaptic currents^9^, we reasoned the neuropil calcium signal from excitatory neurons could provide a good proxy for the LFP *in vivo*. Since the optical signal recorded from specific neuronal populations may reflect the activation of slightly diverse and partially overlapping groups of neurons recorded with the LFP, the effect of anesthesia on those signals could be different, and therefore change their correlations. In addition, the spatial distributions of the neurons generating the two signals are different since the two-photon imaging records the activity from a single plane (with a thickness of a few microns) while the LFP reports the electrical activity originated from cells in a hundred of microns virtual sphere around the electrode, depending on the experimental configuration (e.g. relative distance between the electrode and the reference). This difference might also contribute to brain state-dependent changes in their correlations.

Here we evaluated the extent to which the 2P calcium signal from the neuropil in layer 2/3 could be used to determine the brain state. To this aim, we performed simultaneous *in vivo* two-photon calcium imaging and LFP recordings in layer 2/3 of the somatosensory cortex from Thy1-GCaMP6f mice, which express the calcium indicator GCaMP6f selectively in excitatory neurons^11^. Exploiting this model, we characterized the correlation of the LFP and calcium signals and evaluated its dependence on the anesthesia level. Also, we assessed how reliable is the 2P calcium signal to detect Up and Down states and assess the brain state via the neuropil signal alone. Finally, simulation of the neuropil 2P calcium and LFP signals with a topological spiking network model of AdEx neurons explored the origin of the brain state-dependent correlations.

## Results

### Simultaneous electrophysiological recordings and calcium imaging

**Figure 1:**
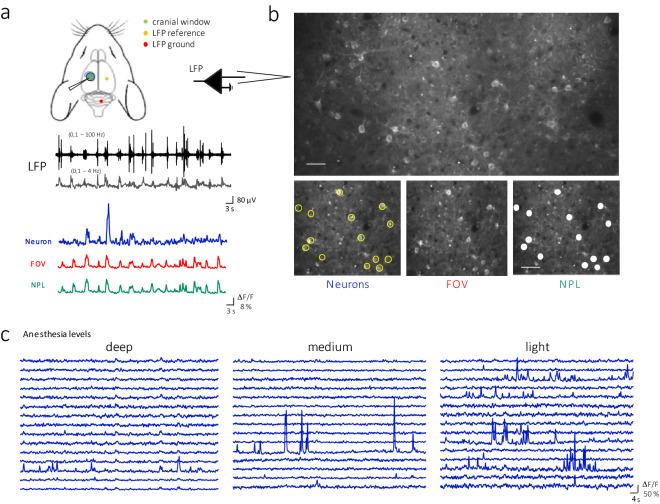
**Simultaneous electrophysiological and two-photon imaging recording of L2/3 excitatory neurons activity in GCaMP6f mice. a. Top.** Experimental scheme. **Bottom.** Representative traces showing the same temporal segment for LFP (black, delta band in grey), Neurons (blue), FOV (red), and NPL (green). **b. Top.** Representative image of two-photon recording of L2/3 excitatory neurons in somatosensory cortex. Scale bar = 30 µm. **Bottom.** Representative ROIs used to extract the calcium dynamics signal from the Neurons (yellow circles), the entire Field Of View (FOV), and the Neuropil (NPL, FOV - Neurons). Scale bar = 45 µm. **c. **Representative time series extracted from 15 neurons in different levels of anesthesia (in this example, corresponding to decreasing percentages of isoflurane concentration).

The local field potential (LFP) and the calcium signal of the somatosensory cortex were recorded simultaneously in n=8 Thy1-GCaMP6f mice anesthetized with different concentrations of isoflurane. The two-photon calcium recordings were performed in an area of $600x300 \mu m^2$ over the primary somatosensory (S1) cortical area, with a resolution of 1024*x*512 pixels. The LFP signal was obtained with a pipette loaded with 1mM Rhodamine dye placed at the edge of the calcium imaging area at a depth of 254.6 ± 90.1 $\mu m$ (average across 67 recordings), that is, at the level of the superficial layers (*Figure* 1).

### Measuring the correlation between two-photon and LFP recordings

**Figure 2:**
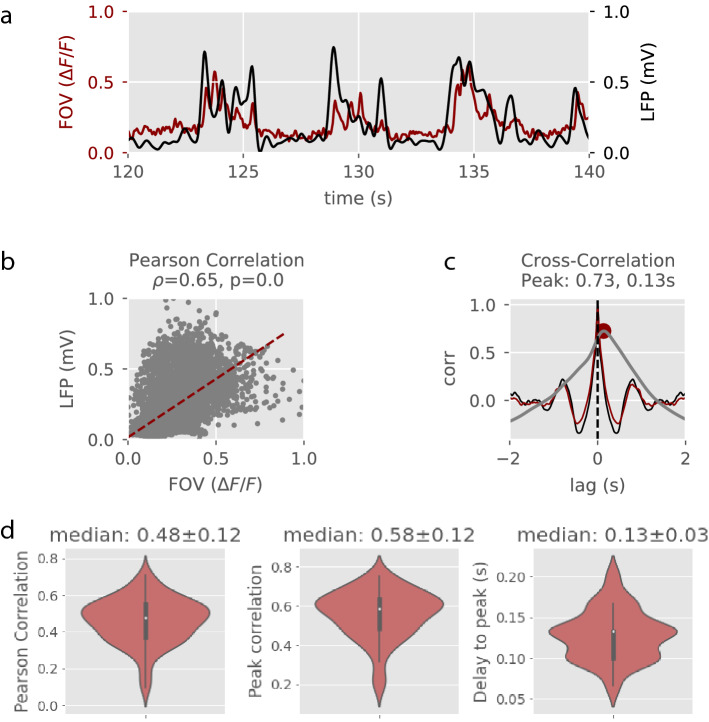
**Correlation measures. a.** Two-photon and LFP normalized signals during 20 seconds of an example recording at 1.5% isoflurane anesthesia. **b.** Pearson’s correlation. **c.** Auto- and cross-correlation of LFP (black trace) and FOV (red trace) signals. The cross-correlation (grey trace) peak is depicted with a red dot. **d.** From left to right, median values of the Pearson’s correlation, the cross-correlation peak and delay to the cross-correlation peak across n = 67 recordings (8 mice).

In order to demonstrate that we can use either the LFP or the calcium signals to assess the brain state we first computed the correlation between the two-photon calcium signal resulting from the average over all the pixels, the Field of View (FOV), and the LFP signal in each recording (*Figure* 2). At the population level (n=8 mice; 67 recordings), LFP and FOV signals were highly correlated: Pearson correlation was 0.48 ± 0.12 on average and the median peak correlation was 0.58 ± 0.12. Interestingly, the calcium signal was delayed with respect to the LFP signal by 0.13 ± 0.03 seconds on average (*Figure* 2).

**Figure 3:**
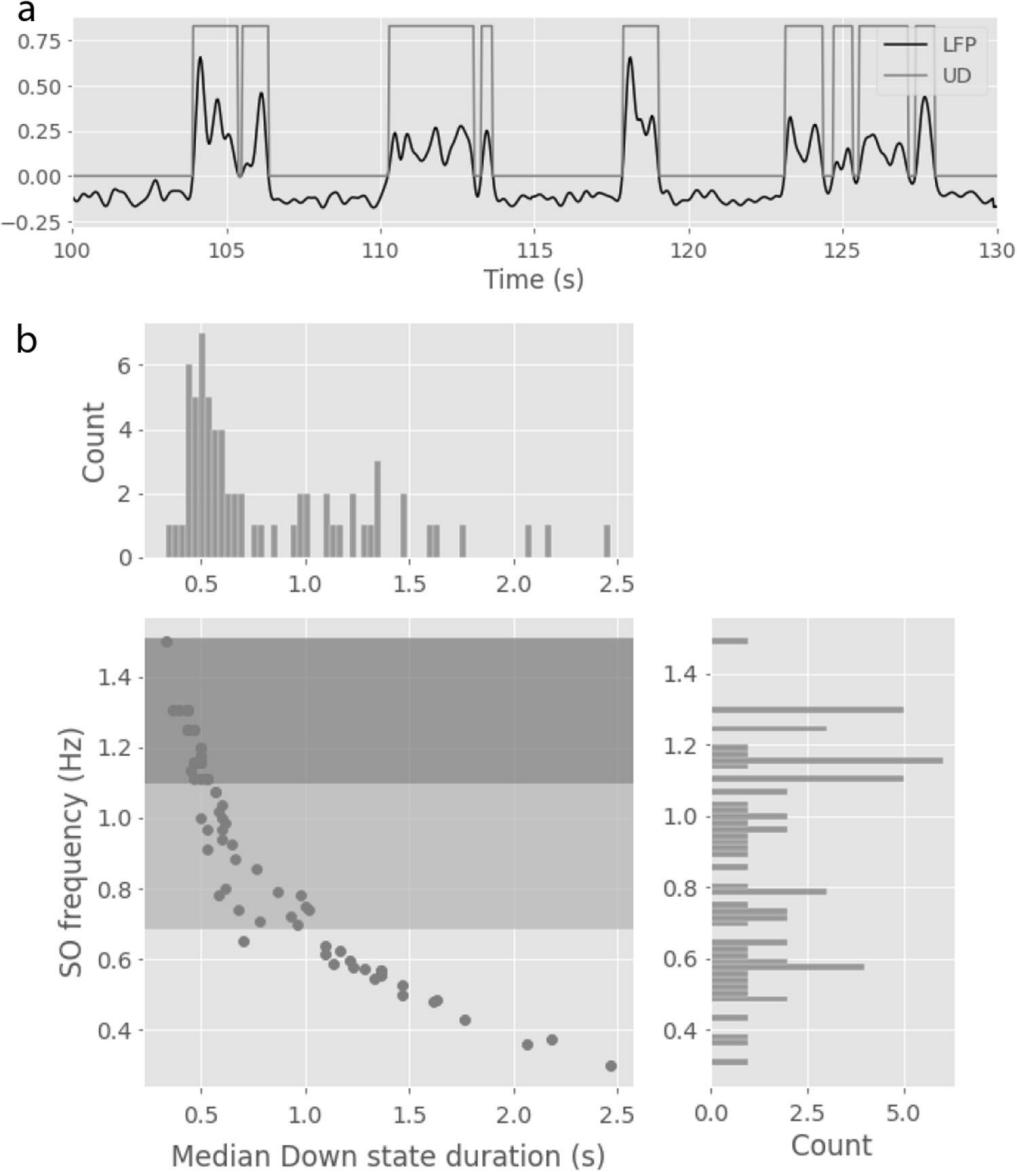
**Up and Down state dynamics in the LFP.** **a.** Example of Up and Down state detection in a 30 seconds recording using the normalized LFP signal (arbitrary units). **b.** Relationship between SO frequency and Down state duration across recordings. Definition of three groups of recordings of decreasing slow oscillation frequency. Data from 67 recordings (8 mice).

Despite the presence of this delay in all recordings, we observed that the distribution of delay values was not uniform. We hypothesised that different depths of anesthesia, that produce a distinct level of syncrhonization of the cortical network, could lead to different delays. To check this hypothesis we wanted to classify the recordings by the level of anesthesia at which they were obtained. The different isoflurane doses administered to the mice in order to achieve different levels of anesthesia largely varied across subjects. A more reliable way to determine the level of anesthesia in electrophysiological data consists in computing the length of the Down states or the frequency of the slow oscillation^12,37^. Therefore, we detected Up and Down states from the LFP signal in each recording and defined three groups of recordings based on the frequency of the slow oscillation (*Figure* 3). We computed the frequency of the slow oscillation in each recording as the inverse of the sum of the median duration of Up and Down states. According to this classification, the group with faster slow oscillations had 23 recordings, the group of intermediate slow oscillation frequency had 24 recordings and the group with slower slow oscillation frequency had 20 recordings.

**Figure 4:**
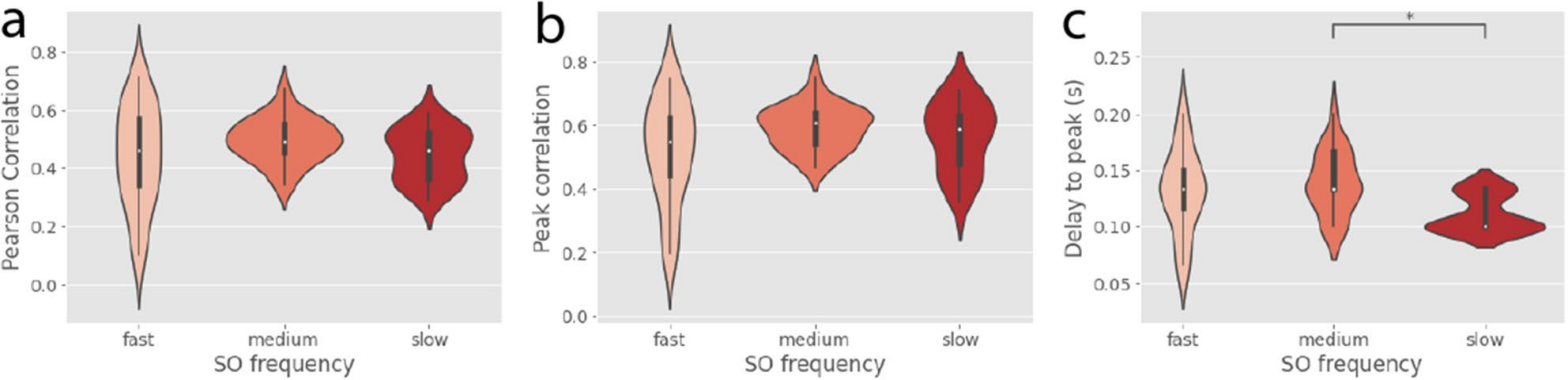
**Correlation between LFP and calcium signals.** LFP-FOV Pearson’s correlation (**a**), peak cross-correlation (**b**) and delay to peak (**c**) as a function of slow oscillation frequency (n=67 recordings, 8 mice).

After classifying the recordings in three groups of decreasing slow oscillation frequency (which corresponds to deeper anesthesia) we observed that the average correlation between the LFP and the FOV signals did not change with the level of anesthesia. On the contrary, the delay between the LFP and the two-photon signal was significantly smaller for deeper anesthesia (*Figure* 4). In any case, in the deeper anesthesia group, the median delay was still 100ms, and we wondered if this delay could be originated by the nature of the FOV signal, compared to the nature of the LFP: while the LFP records the synaptic activity, which reflects the “electric input” to the local network, the FOV signal includes both the synaptic activity arriving to the cells and the calcium spikes of the recorded units, reflecting both the “calcium input” and the “calcium output”. To check if the delay could be produced by the time needed for the cells to integrate the inputs and spike, we decided to compute the neuropil (NPL) signal by subtracting the two-photon activity of each individual cell identified in the imaged area from the FOV signal (*Figure* 1).

**Figure 5:**
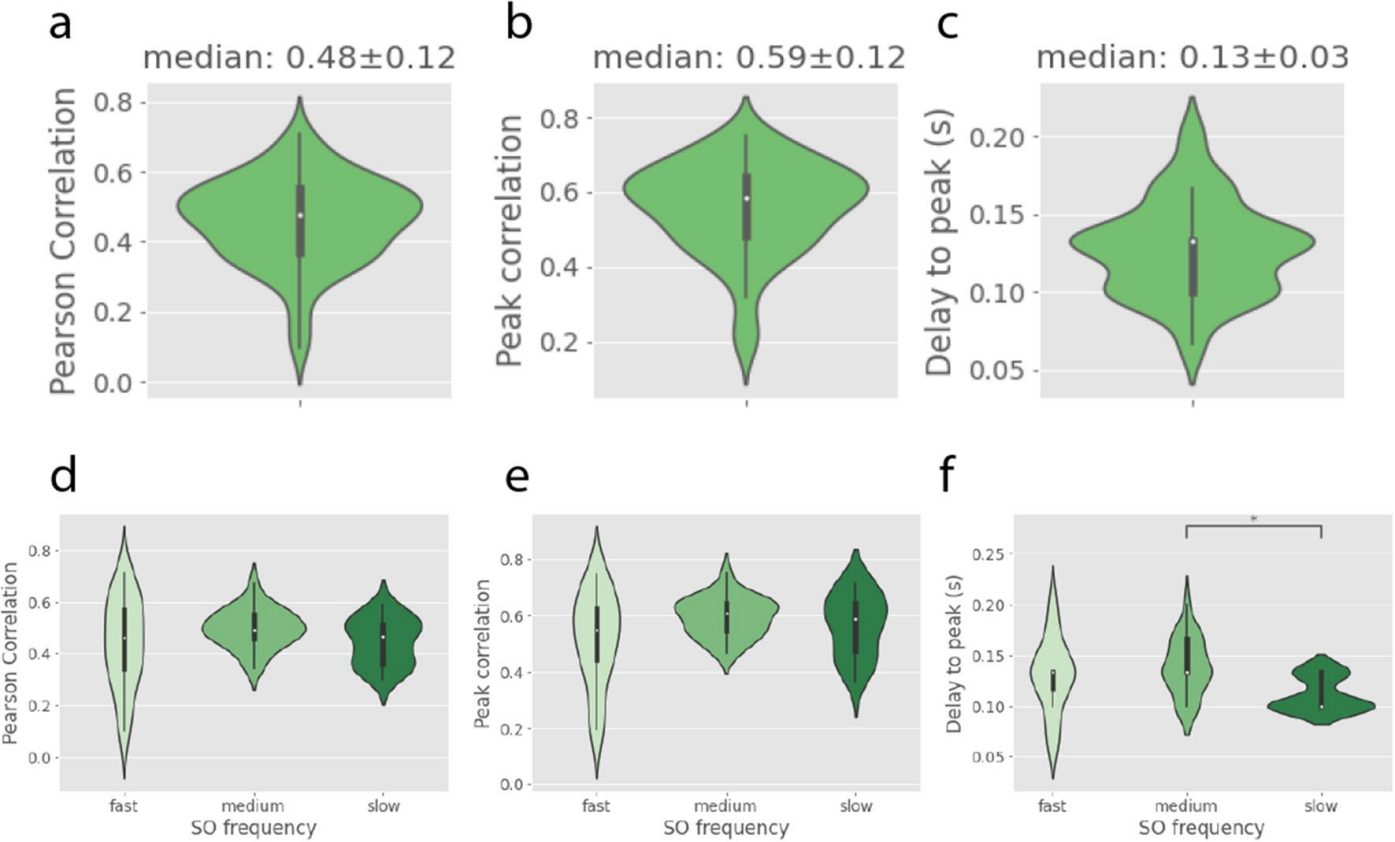
**LFP-NPL correlation measures. a-c.** Median values of the Pearson’s correlation, Peak cross-correlation and Delay to the cross-correlation peak across n=67 recordings (8 mice). ** d-f.** Same correlation measures plotted across different levels of anesthesia.

Then, we computed the Pearson correlation and the cross-correlation between LFP and NPL signals for each recording, as we had done for the FOV signal (*Figure* 2). The population averages (n=8 mice; 67 recordings) show that LFP and NPL signals were highly correlated as well: Pearson correlation was 0.48 ± 0.12 on average and the median peak correlation was 0.59 ± 0.12. Again, the NPL calcium signal was delayed with respect to the LFP signal by 0.13 ± 0.03 seconds on average (*Figure* 5).

Finally, we plotted the values of Pearson’s correlation, peak cross-correlation and delay to peak against the level of anesthesia for the NPL signal, using the previous classification of recordings in 3 groups of decreasing slow oscillation frequency. As in the case of the FOV signal, the correlation between the LFP and the NPL signals remained high regardless of the level of anesthesia, and the delay to peak showed a significant reduction towards deeper anesthesia (*Figure* 5, last row).

### Explaining the delay with a topological spiking network model of AdEx neurons

From the results shown, we conclude on one hand, that the FOV signal correlates well with the LFP signal and that this correlation is independent of the brain state. On the other hand, we have observed that the FOV signal is delayed with respect to the LFP signal on average by 130 ms. This delay is not due to the fact that the FOV signal contains cell’s activity and the delay is brain state dependent, finding the smallest delays in deep anesthesia.

Since the LFP pippete is inserted *besides* the FOV region, the network recorded by the LFP could be slightly different to the one imaged through the two-photon scanner. We hypothesised that this mismatch in the neuronal populations recorded by each technique would produce a delay between signals, and the smaller delay found at deeper anesthesia levels could be explained by the increased global cortical synchrony induced by the anesthesia^6^.

**Figure 6:**
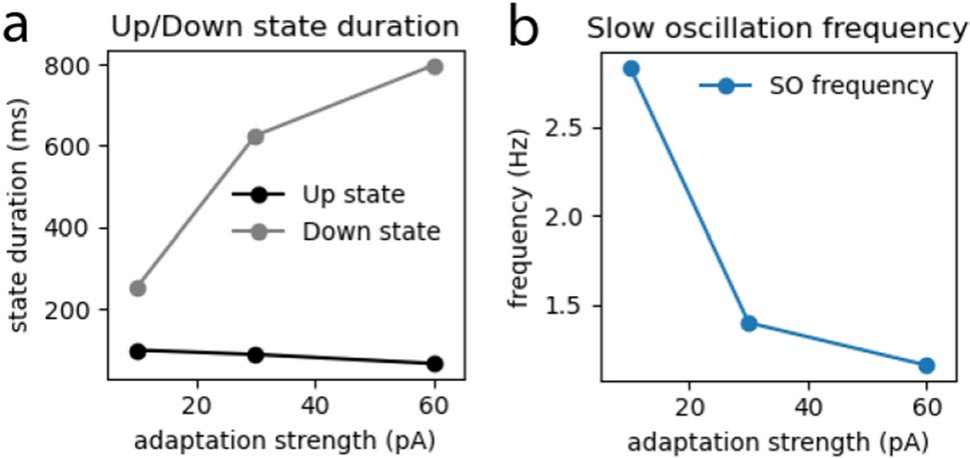
**Slow oscillation measures at increasing adaptation strengths:** Up and Down state duration (**a**) and frequency of the slow oscillation (**b**) at increasing adaptation strenght (parameter b in the model).

To evaluate this hypothesis we used a topological spiking network of 10k adaptive exponential integrate and fire (*AdEx*) neurons^8^ randomly placed in a 2D area of $1700x600\mu m$ with a distance-dependent rule for connectivity (see Methods section). We simulated the spontaneous activity of a cortical network at three different levels of anesthesia by increasing the adaptation strength that affects excitatory neurons only, and that is related to the acetylcholine neuromodulation responsible for the loss of consciousness during sleep or anesthesia^2,23,37^. Increasing the level of adaptation (from $b = 10 pA$ to 30*pA* and 60*pA*) produced slow oscillations with shorter Up states, longer Down states and lower frequency (*Figure* 6).

**Figure 7:**
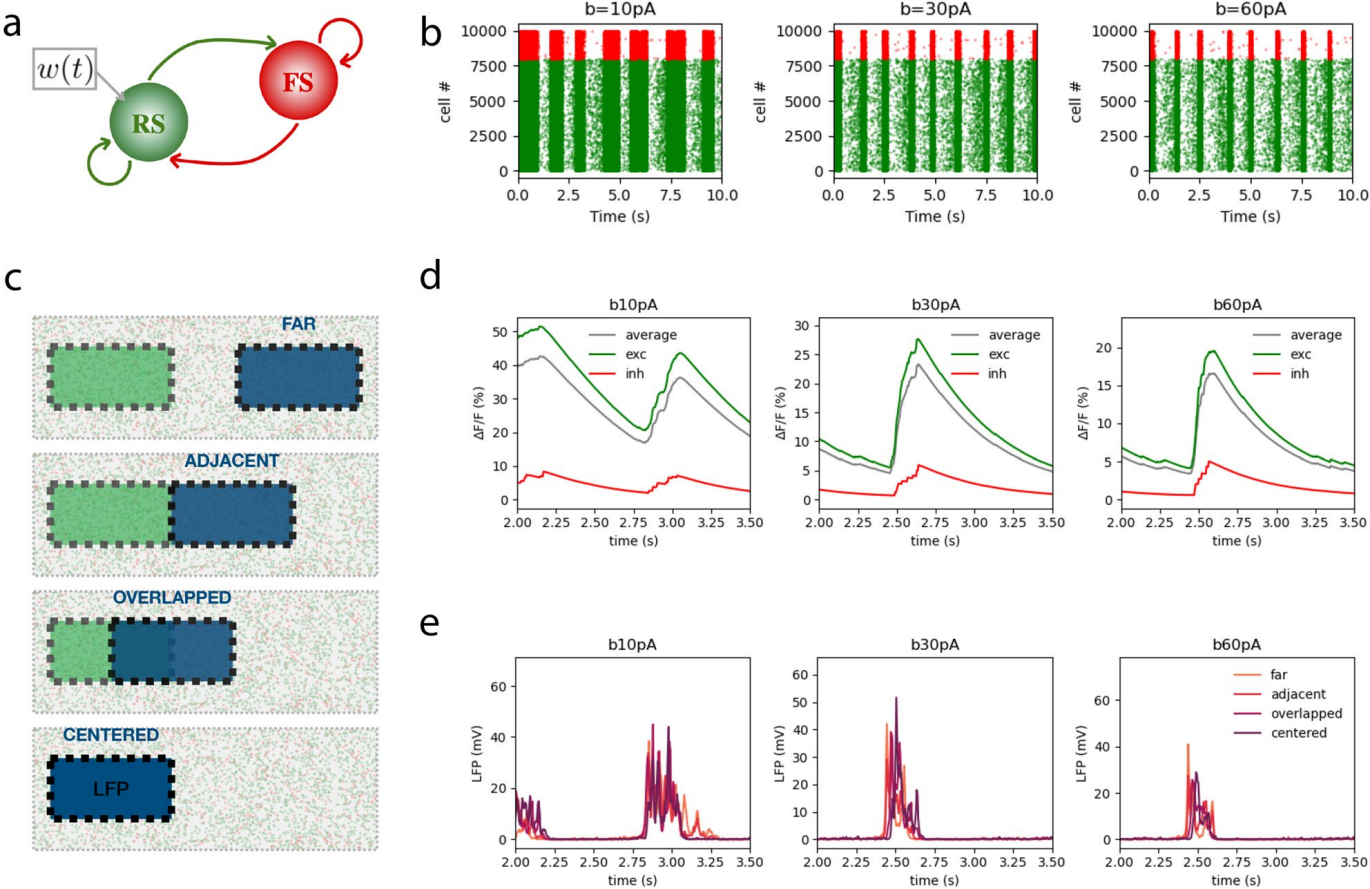
**Simulations of calcium and LFP signals:** **a.** The spiking network model has two populations: one of fast spiking (FS) inhibitory neurons and another of regular spiking (RS) excitatory neurons, which are affected by adaptation ($\omega (t)$). **b.** Raster plots of the spikes of each population (red for inhibitory cells and green for excitatory cells) simulated under increasing adaptation strength (from left to right the strength of adaptation is $b=10, 30$ and 60*pA*, respectively). **c.** 2D plane in which the 10k simulated neurons are located and position of the LFP area (blue) relative to the calcium area (FOV, green) in each condition (the distance between centers in the *centered*, *overlapped*, *adjacent* and *far* conditions are 0, 300, 600 and $900 \mu m$, respectively). Dimensions of the total area: $1700x600\mu m^2$; FOV and LFP areas: $600x300\mu m^2$ **d.** Calcium signal of the excitatory (green), inhibitory (red) and average (grey) populations, simulated under increasing adaptation strength as in b. **e.** LFP signal in each condition in c, simulated under increasing adaptation strength as in b.

In each simulation, we computed the calcium signal from 160 excitatory and 40 inhibitory neurons located within a fixed $600x300\mu m$ area of the network (*Figure* 7). As in^2^, both the membrane potential and the spikes obtained in the simulations were used to compute the calcium currents, the calcium concentration and finally the calcium fluorescence for each population.

For the LFP computation, we considered all the neurons located at different 600x300$\mu$m areas at increasing distances from the calcium area (see *Figure* 7C). For each condition (*centered*, *overlapped*, *adjacent* and *far* for 0, 300, 600 and 900 $\mu m$ between the FOV and the LFP areas, respectively), the spikes of each neuron in each population were convolved with a kernel to obtain the unitary LFP that would be recorded at the center of the LFP area using the method described in^35^.

**Figure 8:**
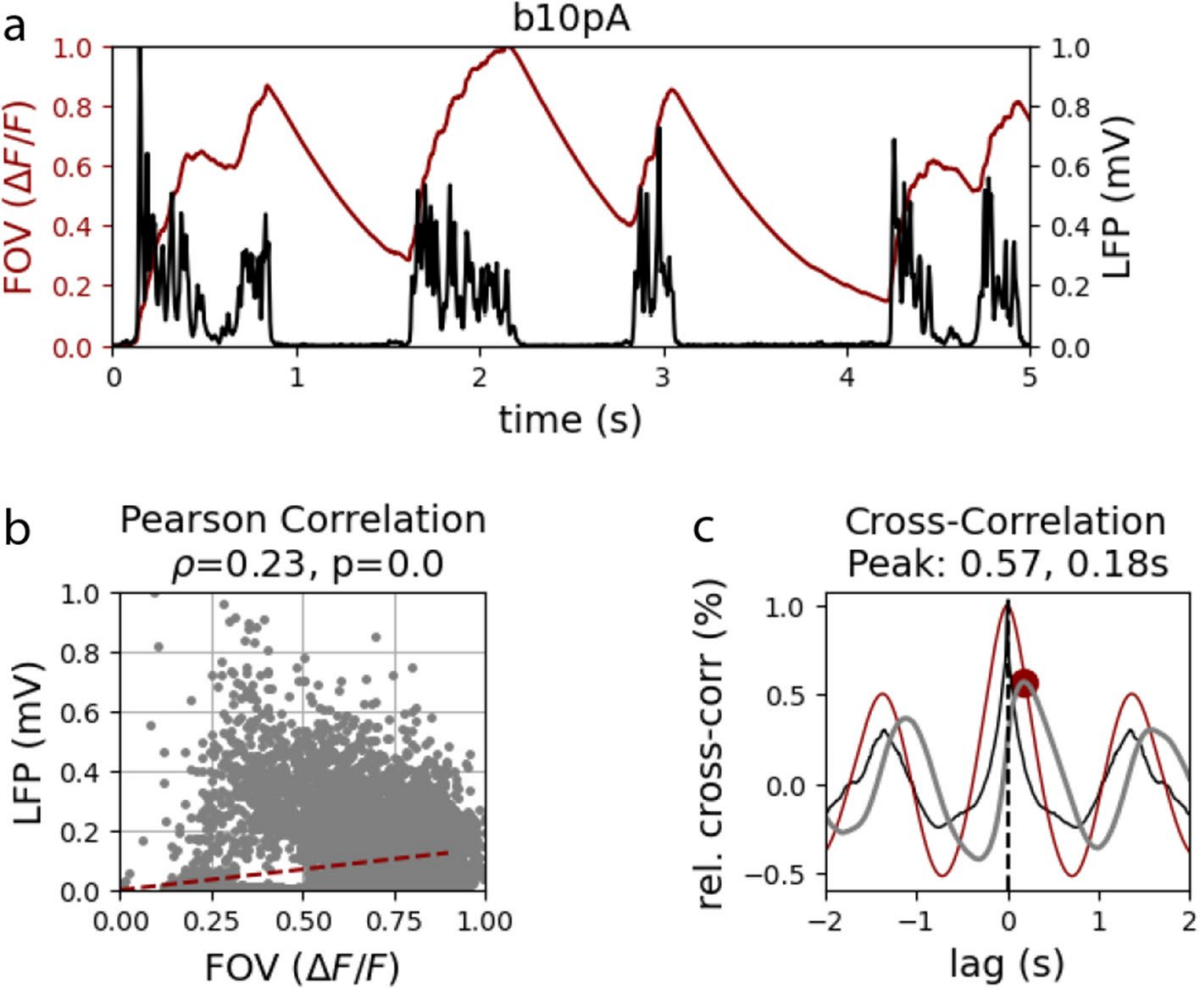
**Correlation measures in the model. a.** Simulated calcium and LFP normalized signals during 20 seconds of simulation at $b = 10 pA$ (light anesthesia) in the *centered* condition. **b.** Pearson’s correlation. **c.** Auto- and cross-correlation of LFP (black trace) and calcium (red trace) signals. The cross-correlation (grey trace) peak is depicted with a red dot.

For simplicity, we considered the averaged signal of the excitatory and inhibitory populations, for both the calcium and the LFP signals. We then computed the Pearson’s correlation and the cross-correlation between the two signals in each condition (*centered*, *overlapped*, *adjacent* and *far*) and in each adaptation strength as we had done for the experimental data (*Figure* 8).

**Figure 9:**
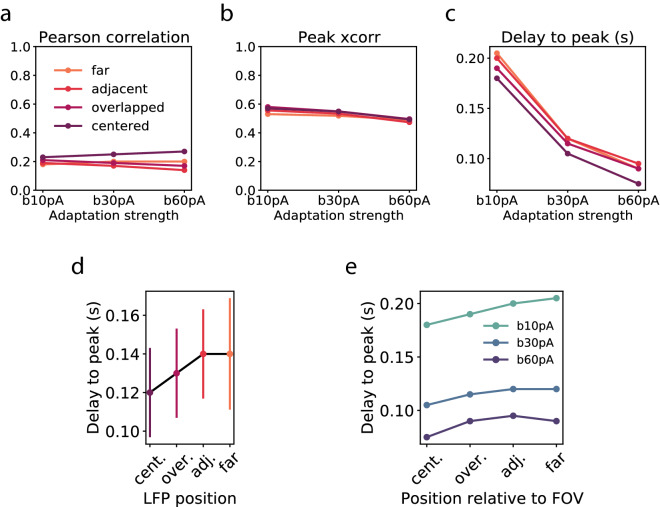
**Correlation between calcium and LFP for each condition. a-c.** Pearson’s correlation, peak cross-correlation and delay to peak as a function of the adaptation strength. Colors indicate the condition, that is the position of the LFP area with respect to the calcium area. **d.** Average across different adaptation strengths of the delay to peak as a function of the condition. Error bars indicate the standard error of the mean. **e.** In each adaptation strength, delay to peak as a function of the condition.

As shown in *Figure* 9, the model was able to reproduce the fact that the calcium and LFP signals were correlated independently of the brain state in all the conditions: 0.2 ± 0.03 Pearson’s correlation and 0.53 ± 0.03 peak correlation on average (across conditions and brain states). Moreover, the model also showed a delay between the LFP and the calcium signal that decreased with the strength of adaptation in all conditions, with an average value of 0.13 ± 0.05 seconds. Importantly, for each brain state we observed that the delay increased with the distance between calcium and LFP regions, finding the largest delay in the *far* condition, in which the LFP and the calcium signals are computed from very distant populations of neurons. We found the same result averaging across the different adaptation strengths. Therefore, we conclude that the delay is due to the fact that the calcium and the LFP signals come from slightly different regions of the brain and that when the brain activity is more synchronous this delay is reduced without affecting the correlation value.

### Up and Down states detection

An important feature for assessing the level of consciousness is the presence or absence of slow oscillations, and if present, the length of Up and Down states and the frequency of the slow oscillation^12,37^.

These relevant measures depend on the accuracy of the detection of Up and Down states, usually performed on electrophysiological signals such as the LFP. Here we want to test the suitability of calcium signals for Up and Down states detection and therefore demonstrate that it is possible to assess the level of consciousness with calcium imaging techniques.

**Figure 10:**
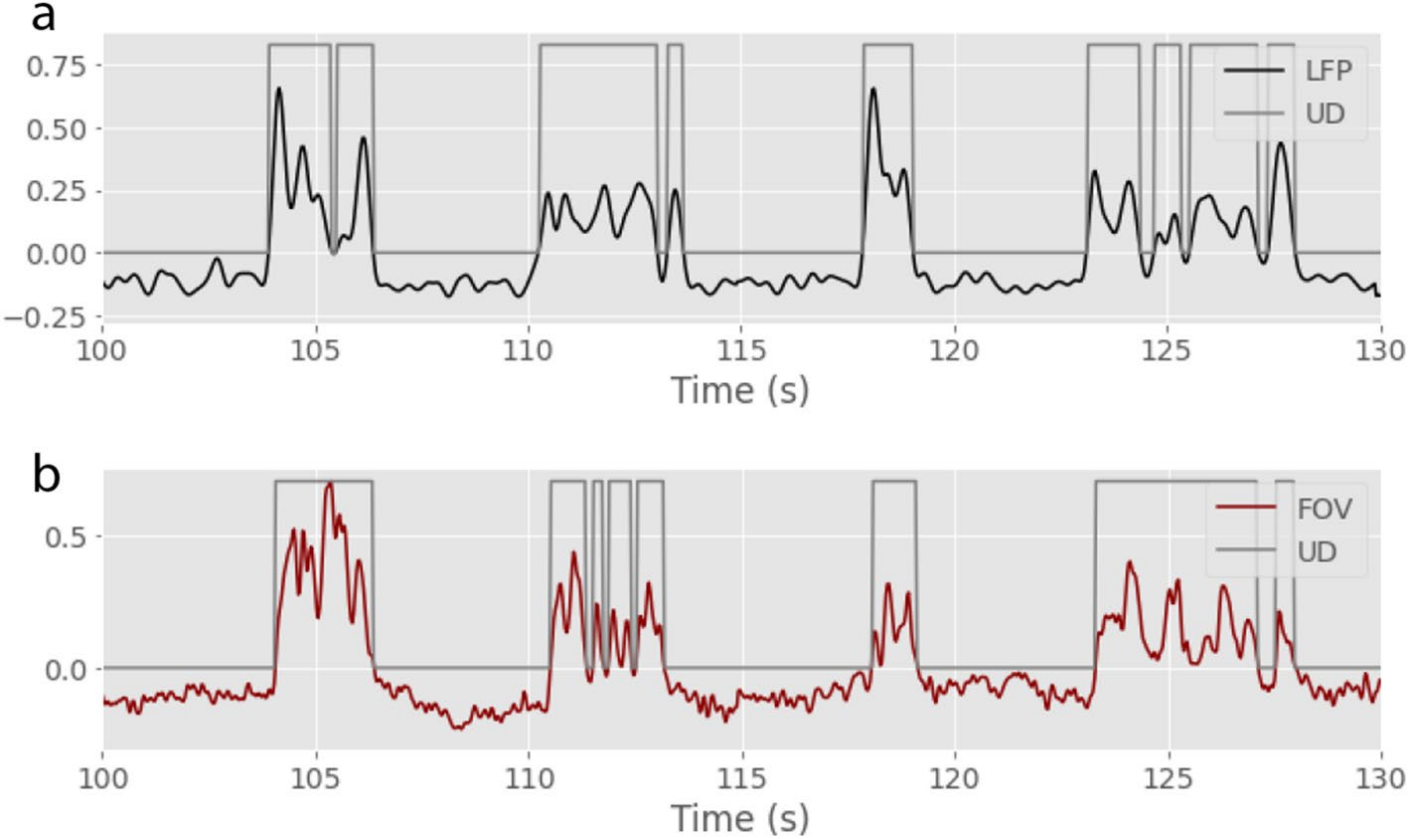
**Up and Down state detection in LFP and calcium signals.** Example of Up and Down state detection in a 30 seconds recording using the LFP (**a**) or the FOV (**b**) signals. .

We used the same algorithm (see Methods section) to detect Up and Down states from LFP and FOV signals in each recording (*Figure* 10).

**Figure 11:**
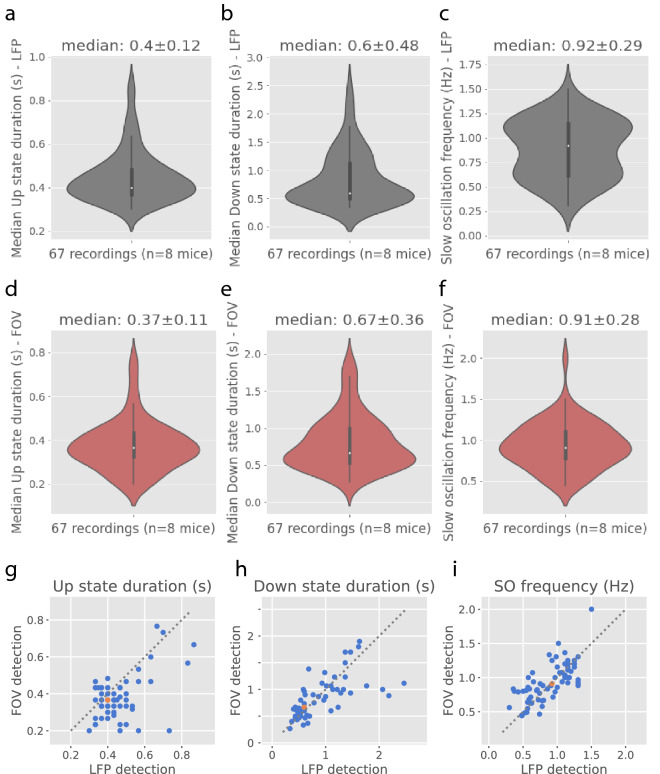
**Up and Down state detection from FOV or LFP signals** From left to right, Up state duration, Down state duration and frequency of the slow oscillation detected with the LFP signal (**a-c.**) or the FOV signal (**d-f.**). **g-i.** Correspondance between LFP and FOV measures shown graphically. Each blue dot corresponds to a different recording. The orange dot is the median. The grey dashed line is the model x=y.

We found that the median duration of Up or Down states, as well as the median frequency of the slow oscillation detected with either the LFP or the FOV signal are practically the same: $0.40\pm 0.12$ s *versus* $0.37\pm 0.11$ s for the median Up state duration; $0.60\pm 0.48$ s *versus* $0.67\pm 0.36$ s for the median Down state duration and $0.92\pm 0.29$ Hz *versus* $0.91\pm 0.28$ Hz for the median slow oscillation frequency, using the LFP or the FOV signals, respectively (*Figure* 11).

## Discussion

In this paper, we have explored whether the calcium signals from two-photon (2P) calcium imaging can be used to assess brain state, in this case, the level of anesthesia. In particular, we focused on either the wide field signal (FOV) or the signal from the neuropil (NPL, removing somata from FOV). By measuring these signals simultaneously with a microelectrode for LFP recording, we found a high correlation between LFP and FOV/NPL. We further showed that this level of correlation did not change with the level of anesthesia, which is good evidence that the correlation is not due to network activity but stems from a real overlap between the two signals. However, we also found that the delay between the LFP and the two-photon signal (both FOV and NLP) was dependent on brain state, and was significantly smaller for deeper anesthesia. Using computational models, we could reproduce this result, predicting that the delay is due to the fact that the calcium and the LFP signals come from slightly different regions of the brain. When brain activity is more synchronous, this delay is reduced without affecting the correlation value. Thus, calcium signals appear to be suitable to assess brain state, and perform Up and Down states detection (which is difficult with 2P data from cellular spiking activity). In the following, we discuss the significance of these main findings, relate them to previous work, and outline perspectives that they offer for future work.

One of the very first study to combine LFP recordings with 2P calcium imaging was done by^19^ in an attempt to measure the “input” and “output” of neocortical networks *in vivo*. In that study, the authors reported that the neuropil signal, which they called “Optical Encephalogram” (OEG), is highly correlated with the surface potential, the electro-corticogram (ECoG). This is in full agreement with the measurements we provide here, where the LFP is found to be highly correlated to the neuropil signal generated by excitatory neurons. As the neuropil signal results from a combination of synaptic calcium signals in dendrites, action potentials in axons, and glia, the authors qualified the OEG as providing information about the “input” of the network. In the framework of assessing brain state, we suggest here that the calcium signal from excitatory neurons also provides a measurement of the distributed activity similar to the LFP, which is thought to be dominated by synaptic currents ^9^.

It is important to note that it was found that LFP signals contain a strong contribution from inhibitory postsynaptic currents (IPSCs) in human and monkey^34^. This conclusion was reached by calculating the relationship between the 96 LFP electrodes in Utah-array recordings, with single-unit activity of regular-spiking (RS) and fast-spiking (FS) neurons. FS cells produce the strongest unitary fields compared to RS cells, corroborating previous studies in hippocampal slices^5,14^. Our measurements do not provide information about a possible dominance of IPSCs in LFP or neuropil activity, but we expect that IPSCs do contribute to the neuropil and FOV signals, as they participate to sculpt the voltage activity in dendrites. Thus, the correlation we find between LFP and field of view or neuropil signals could be due, in part, to the postsynaptic contribution of inhibitory neurons.

More recent work by^7^ also used 2P calcium imaging, to show that Layer 5 pyramidal neurons strongly synchronize during slow oscillations during anesthesia. The present recordings were done in superficial layers (essentially Layer 2/3), where we found that the neuropil and FOV signals correlate with the LFP oscillations during anesthesia. As the calcium signal was due to pyramidal cells in our preparation, the neuropil signal presumably contains contributions from all dendrites in superficial layers, which include Layer 2/3 pyramidal cell dendrites, but also deep layer pyramidal cell apical dendrites. Thus the correlations we find are also in agreement with this recent study.

One of our main findings is that the correlation between LFP and the calcium signal did not depend on brain state, which suggests that both the neuropil and FOV reflect information similar to the LFP, and thus can be used to infer the state of the brain similarly to the LFP.

However, we also found that there is a substantial delay between the two signals, which can be in 100 ms range. Even though calcium dynamics are used as a proxy for neuronal spiking activity, biophysical constraints make calcium dynamics significantly slower than membrane potential fluctuations^3,32^. Indeed, ionic diffusion/extrusion and intrinsic and extrinsic calcium buffers result in complicated interactions that affect calcium kinetics^22^. Interestingly, the intrinsic delay between electrophysiological recordings and GCaMP6f signal has been reported to be $\sim$40 ms both at single cell level^10^ and at the population level^20^. Thus we considered this delay as a fixed component of the delay we observed. Despite this static component which is an intrinsic property of the calcium measure, here we found a brain-state dependent component of the delay.

We propose two different methods for evaluating the level of correspondence between electrophysiological and calcium imaging signals. On the one hand, we measure the cross-correlation peak between the signals, and the delay to this peak. On the other hand, we first detect Up and Down states and then measure some characteristic properties of the slow oscillation. Of course, the second method can only be used in the presence of slow oscillations, and in consequence, this methodology would not be indicated in the case of brain states in which the activity is desynchronized, such as quiet wakefulness, running, or some phases of natural sleep.

We provided a computational model to explain these results where we took into account the fact that the LFP electrode was located outside of the imaged field. The model predicts that the delay is due to this spatial separation, and that the state dependence of the delay can be accounted for by the different levels of synchrony of different brain states. But most importantly, the model could reproduce the state independence of the correlations between the calcium and LFP signals. In future work, it would be interesting to further check these model predictions by using more realistic models of the cortical tissue, where the various cellular processes (soma, dendrites, axons, glial cells) are explicitly integrated for a more quantitative match with the experimental measurements.

Recently, the development of transparent graphene microelectrode technology enabling crosstalk-free integration of 2P microscopy, optogenetics, and cortical recordings in vivo will represent a useful tool for dissecting the correlation of the electrical and optical signal over a larger spatial scale^36^. Finally, a perspective would be to follow a similar approach at a larger scale, by relating signals such as the wide-field calcium signal, with large-scale electric signals such as the EcoG or the electro-encephalogram (EEG) (e.g. as in^16,38^). The present observation that the field-of-view signal and neuropil signal largely overlap, shows that the contribution of somata is comparatively small. A consequence is that wide-field calcium signals measured from the brain surface, are perhaps also dominated by synaptic currents and should thus highly correlate with EcoG or EEG signals. This should be examined by future studies where these signals could be recorded simultaneously. Last, extending the present study on slow-wave activity to other physiological states like running, resting, and natural sleep, will be necessary to understand possible changes in the correlation of the calcium and LFP signals in awake subjects.

## Materials and methods

### Experimental recordings

All experimental procedures were performed in accordance with the ARRIVE guidelines (https://arriveguidelines.org), the directive 2010/63/EU on the protection of animals used for scientific purposes and approved by the Italian Minister of Health, authorization n. 723/2019. We recorded the cortical activity from n = 8 Thy1-GCaMP6f mice (RRID:IMSR_JAX:025393;^11^); 4 females and 4 males with average weights 35±3 g and 40±4 g, respectively.

For the surgery, mice were deeply anesthetized with isoflurane. The protocol followed for the cranial window preparation was slightly modified from^17^ and^21^. Briefly, we administered anesthetized mice a subcutaneous injection of dexamethasone (0.04 mL per 2 mg/ml). The animals were then placed into a stereotaxic apparatus and after applying the local anesthetic lidocaine 2% (20 mg/ml), the skin over the skull was removed. Using a dental drill, the border of a 3-mm-wide circular area above the primary somatosensory cortex (AP -2 mm, ML +2 mm from bregma) was thinned and the central part of the bone was gently removed. The exposed brain was covered with a cover glass, leaving a small portion of the window open for the LFP electrode to be inserted. The optical window was sealed to the skull with a mixture of dental cement and acrylic glue. The exposed skull was covered using dental cement (Super Bond, C &S, Sun medical Moriyama City, Shiga, Japan). The surgery was followed by the imaging session under the two-photon microscope. To induce different brain states we used different doses of isoflurane anesthesia. The commercial apparatus for two-photon microscopy (Bergamo II Series Multiphoton Microscope, Thorlabs) was combined with a mode-locked Ti: Sapphire laser (Chameleon, Coherent Inc.) to supply the excitation light. The laser beam was scanned in the xy plane by a galvo-resonant system. An objective lens (XLUM 20X, NA 0.95, WD 2 mm, Olympus) focused the beam onto the specimen. A closed-loop piezoelectric stage allowed axial displacements of the objective.

To record the LFP signals, we inserted a pipette filled with 1mM Rhodamine dye at the edge of the imaging area. LFP was recorded using a borosilicate glass microelectrode (8-10M$\Omega$), backfilled with a filtered solution of 2M NaCl and 0.1mM sulforhodamine 101 (S76352, Sigma-Aldrich) to allow the electrode tip visualization. The microelectrode was placed under visual guidance into the cortical layer 2/3, coplanar with the imaging depth. A reference screw was placed on the contralateral parietal bone and a ground screw was placed on the occipital bone.

Voltage signals were recorded using a 3000 AC/DC differential amplifier, sampled at 10 kHz, high-pass filtered at 0.1 Hz, low-pass filtered at 3kHz and digitized at 10kHz using a USB-621 interface (National Instruments).

After the imaging session, all animals were perfused with 150ml of paraformaldehyde 4% (PFA, Aldrich, St. Louis, Missouri, USA).

### Signal synchronization

A file containing the triggers for the calcium recordings (signalling the onset of each two-photon scan) was sampled at the same sampling frequency as the the LFP signal (10KHz). Using these triggers we aligned the LFP and calcium signals, and downsampled the LFP at the same sampling rate as the two-photon signals (30 Hz), by averaging the LFP signal between two consecutive two-photon samples.

### Neuropil computation

To compute the neuropil (NPL) signal from the calcium signals in each recording, the somas of the cells were identified, and the average activity of the pixels corresponding to the soma area was saved independently for each cell. Then, the activity of all the cells was subtracted from the Field of View (FOV) signal, that is the average signal from all the pixels (1025x512 pixels).

### Signal processing

The signals were processed to filter out the low frequency components responsible of the drifts, and the higher frequency components that correspond to noise. The LFP signal was filtered between 10 and 15 Hz. This resulted in a LFP signal that increased with increased activity, independently of the depth of the tip of the electrode. FOV and NPL signals, on the contrary, had more noise in this frequency range, so we filtered them between 0.1 and 1.5Hz. All signals were normalized after filtering.

### Correlation measures

To compare the LFP signals with the calcium signals we used Pearson’s correlation and cross-correlation. We normalized the cross-correlation values by the standard deviation of the two signals. We evaluated the *peak correlation* as the normalized cross-correlation value at the peak and the *zero-lag correlation* as the normalized cross-correlation value at the zero lag which corresponded to the Pearson’s correlation value (not shown). The *delay to peak* was evaluated as the lag at which the cross-correlation peaked.

### Statistics

To compare the different levels of anesthesia we used ANOVA and Tukey’s method for post-hoc testing the significance of the differences between pairs of groups. All the analyses were performed using the module statsmodels in python scripts.

### Up and Down state detection

The normalized signal was sliced in smaller chunks of 15 seconds. For each chunk, the mean was subtracted and the standard deviation (STD) computed. Then a threshold was set at 0.1 times this STD. Periods longer than 80 ms in this 15 second signal that were over the threshold were marked as an Up state.

### Spiking network model

The network contains $N = 10.000$ neurons. 20% of them are inhibitory, fast spiking neurons and the other 80% are excitatory, regular spiking neurons. The neurons are modeled as adaptive exponential integrate and fire (*Adex*) neurons^8^. Including spike frequency adaptation, a network of *Adex* neurons can reproduce both asynchronous or synchronous activity regimes^13^, and therefore is ideal to simulate the cortical dynamics of different levels of consciousness or brain states such as wakefulness, sleep or anesthesia^2,23^. The equations that drive the dynamics of *Adex* neurons are the following:

$$\begin{aligned} C_m \frac{dV}{dt} = -G_l(V-E_l) + G_l\Delta _V\exp ^{(\frac{V-V_{thre}}{\Delta _V})} + I_{syn}+\sigma \xi -\omega \\ \frac{d\omega }{dt} = -\frac{\omega }{\tau _{\omega }} +b\sum _k \delta (t-t_k) + a(V-E_l) \end{aligned}$$

where the synaptic input is defined as:

1 $$\begin{aligned} I_{syn}=\sum _i g_i^{syn}(V-E_i^{syn}) \end{aligned}$$

with

2 $$\begin{aligned} \frac{dg_i^{syn}}{dt}=-g_i^{syn}/\tau _{syn} \end{aligned}$$

As in^2^, the leak conductance is $G_l=10nS$, the membrane capacitance is $C_m=150pF$, the resting potential is $E_l=-60mV$ for excitatory cells and $E_l=-65mV$ for inhibitory cells, the steepness of the exponential approach to threshold is $\Delta _V = 2.0$ and $\Delta _V = 0.5$ for excitatory and inhibitory cells, respectively, the spiking threshold is $V_{thre}=-50mV$, the refractory period is defined by $V_{reset}=-60mV$ and $T_{refrac}=5ms$. The term $\sigma \xi$ in the membrane potential equation represents a gaussian noise of amplitude $\sigma = 50$. The adaptation $\omega$ is modeled with the parameters $a=4nS$ and $\tau _{\omega }=500ms$. To simulate increasing levels of isoflurane anesthesia we used different values of the parameter *b* in the model, that represents the strength of adaptation: at each spike, the adaptation current is incremented by a value *b*.

### Gaussian connectivity

The neurons are connected following a distance-dependent rule and a certain probability of connection that depends on the type of synapse:

3 $$\begin{aligned} p_{ij} = p_0\exp ^{-\frac{(x_i-x_j)^2+(y_i+y_j)^2}{2\sigma ^2}} \end{aligned}$$

where $p_0=0.205, \sigma = 130 \mu m$ for excitatory synapses and $p_0=0.72, \sigma = 110 \mu m$ for inhibitory synapses.

### Calcium model

We used the calcium model described in^2^, where we take into account the dynamics of voltage gated calcium channels to translate the changes in the membrane potential due to the spiking activity into inward current of calcium ions. Then we evaluate the internal concentration of calcium as a function of these currents and the slow decay to the basal concentration of calcium ($\tau _{Ca} = 760 ms$). Finally, we compute the calcium signal (fluorescence) as a function of the internal calcium concentration taking into account the specific calcium indicator (GCamp6f) parameters:

4 $$\begin{aligned} Ca = k_F\frac{[Ca^{2+}]^{n_H}}{[Ca^{2+}]^{n_H}+k_d} \end{aligned}$$

where $K_F = 10$ is a scaling factor, $k_d = 375nM$ is the dissociation time constant for GCaMP6f, a measure of the affinity of the fluorescent indicator to the calcium ion, and $n_H = 2.3$ is the Hill coefficient^10^.

The calcium signal was obtained for the excitatory and inhibitory populations ($Ca_{exc}$ and $Ca_{inh}$, respectively). The average calcium signal (*Ca*) of the network was computed as follows:

5 $$\begin{aligned} Ca = 0.2*Ca_{inh}+0.8*Ca_{exc} \end{aligned}$$

### LFP model

To estimate the LFP from the spiking neurons we use the method described in^35^. We consider the LFP recorded in the center of a squared area of $600x300 \mu m$. The LFP generated by each population is computed as the sum of the unitary LFPs (*uLFP*) obtained from the convolution of the spike train of each neuron with a gaussian kernel:

6 $$\begin{aligned} uLFP(x,t) = A(x)exp^{-\frac{(t-tp)^2}{2\sigma ^2}} \end{aligned}$$

where *A*(*x*) is an amplitude that decays with the distance:

7 $$\begin{aligned} A(x)= A_0exp^{-\frac{|x-x_0|}{\lambda }}, \end{aligned}$$

with the maximal amplitude $A_0=3\mu V$ and $0.48\mu V$ for the inhibitory and the excitatory cells, respectively, as we consider the LFP recorded from the *soma* layer, and the space constant of the decay $\lambda = 0.2mm$. $|x-x_0|$ is the distance between the position of the electrode and the soma of each cell.

$\sigma$ in equation 6 is the standard deviation in time and takes the values 2.1 and 3.15*ms*, for inhibitory and excitatory cells, respectively, and *tp* is the peak time of the uLFP, defined as:

8 $$\begin{aligned} t =t_0 + d + |x - x_0 |/\nu _a , \end{aligned}$$

where $t_0$ is the time of the spike, $d=10.4ms$ is a constant delay and the axonal velocity is $\nu _a=0.2m/s$.

The average LFP over populations was computed as:

9 $$\begin{aligned} LFP = 0.2*LFP_{inh}+0.8*LFP_{exc} \end{aligned}$$

## Data Availability

The datasets used and/or analysed during the current study available from the corresponding author on reasonable request.
